# Rapid Liquid Chromatographic Method for the Determination of Roflumilast in the Presence of Degradation Products

**DOI:** 10.4103/0250-474X.70496

**Published:** 2010

**Authors:** V D Barhate, Priya Deosthalee

**Affiliations:** Department of Chemistry, V. E. S College of Arts, Science and Commerce, Chembur, Mumbai - 400 071, India

**Keywords:** Forced degradation, liquid chromatography, roflumilast, RRHT

## Abstract

A forced degradation study on roflumilast drug substance was conducted under the conditions of hydrolysis, oxidation, thermal and photolysis. The method was developed and optimized by analyzing forcefully degraded samples. The best separation was achieved on a Zorbax SB C18 1.8 µm column with 0.005 M ammonium formate buffer pH 3.5 and acetonitrile as mobile phase in a 13 min run time. The proposed method was able to resolve all the possible degradation products formed during stress study. The drug was stable to neutral, thermal and photolytic conditions but unstable to acidic, alkaline and oxidative conditions at 80° for 24 h. The degradation products resulting from stress study did not interfere in assay and related substances of roflumilast and thus the method can be regarded as stability indicating. An alternate method was also developed on a conventional 250×4.6 mm, 5 µm column wherein runtime was 38 min. Thus rapid resolution high throughput column was able to reduce the run time from 38 min to 13 min.

Roflumilast is a novel, potent, selective phosphodiesterase 4 (PDE4) inhibitor for the treatment of chronic obstructive pulmonary disease (COPD) and asthma (fig. 1). Its empirical formula is C_17_H_14_Cl_2_F_2_N_2_O_3_and its molecular weight is 403.21. There is no reported method for the analysis of roflumilast in the presence of its degradation products. In the absence of an official roflumilast monograph in the pharmacopoeias, including the European Pharmacopoeia, British Pharmacopoeia and United States Pharmacopoeia, development of such a method would prove useful for the industry. The objective of this work was, to develop a simple, economic and rapid HPLC method for the quantitative analysis of roflumilast.

**Fig. 1:**
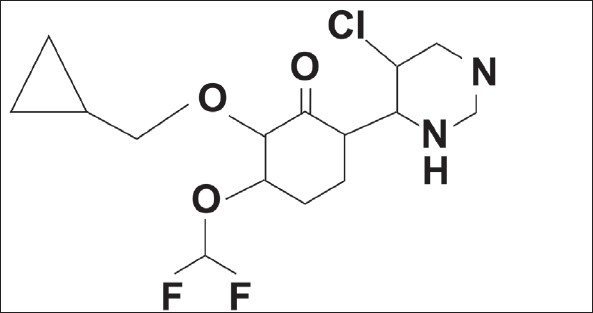
Structure of roflumilast

Roflumilast was received as a gift sample from MSN laboratories limited. Acetonitrile and methanol both (Merck) were of HPLC grade. GR grade ammonium acetate, monobasic sodium monophosphate monohydrate, dibasic sodium phosphate anhydrous, hydrochloric acid, acetic acid glacial, formic acid, ortho phosphoric acid and sodium hydroxide pellets were all procured from Merck India limited. Ammonium hydrogen carbonate and hydrogen peroxide 30%w/v were of (Qualigens) ExcelR and SQ grade, respectively. Ammonium formate was of 99.995+% pure (Sigma Aldrich). HPLC grade water was obtained through milli Q water purification system.

The LC system used for method development and forced degradation studies was Agilent 1100 series liquid chromatographic RRHT (Rapid Resolution High Throughput) system comprising of binary pumps, column oven, autosampler and diode array detector. Data acquisition and processing was carried out using Chemstation software.

The chromatographic separation was achieved using gradient elution (Table 1) consisting of 0.005 M ammonium formate buffer pH 3.5 and acetonitrile on a Zorbax SB C18 50×4.6 mm, 1.8 µm column. Flow rate was maintained as 0.5 ml/min. The column was thermostated at 25° and the injection volume was 3 µl. The UV wavelength of detection used was 215 nm. Roflumilast solution of concentration 150 µg/ml was prepared in acetonitrile and chromatogram is depicted in fig. 2. Roflumilast was stressed under different conditions to promote degradation.

**TABLE 1:** GRADIENT ELUTION PROGRAM

Time (min)	Aqueous phase (%)	Acetonitrile (%)
0	90	10
3	47	53
5	43	57
7	40	60
9	10	90
10	10	90
11	90	10
13	90	10

**Fig. 2:**
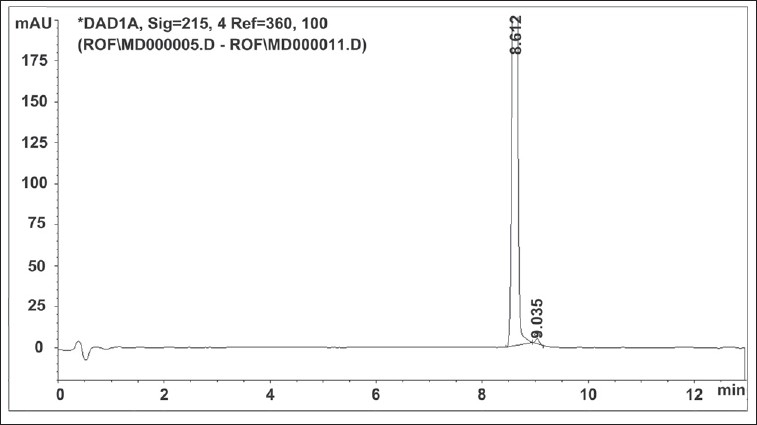
Typical chromatogram of roflumilast sample.

Solution for hydrolytic degradation was prepared by weighing accurately about 15 mg of roflumilast in different 20 ml volumetric flasks. Roflumilast was dissolved in 1 ml of acetonitrile and kept in hot air oven for 24 h at 80° after adding 5 ml of 0.1 N HCl for acid hydrolysis, 5 ml of 0.1 N NaOH for base hydrolysis, 5 ml of water for neutral hydrolysis, individually. After heating, acid hydrolysis solution was neutralized with 0.1 N NaOH and base hydrolysis solution was neutralized with 0.1 N HCl.

Solution for oxidative degradation was prepared by weighing accurately about 15 mg of roflumilast in a 20 ml volumetric flask and dissolving in 1 ml of acetonitrile. The flask was kept in hot air oven for 24 h at 80° after adding 5 ml of 30% hydrogen peroxide. The chromatograms for alkaline, acidic and oxidative degradation are depicted in fig. 3, fig. 4 and fig. 5 respectively.

**Fig. 3:**
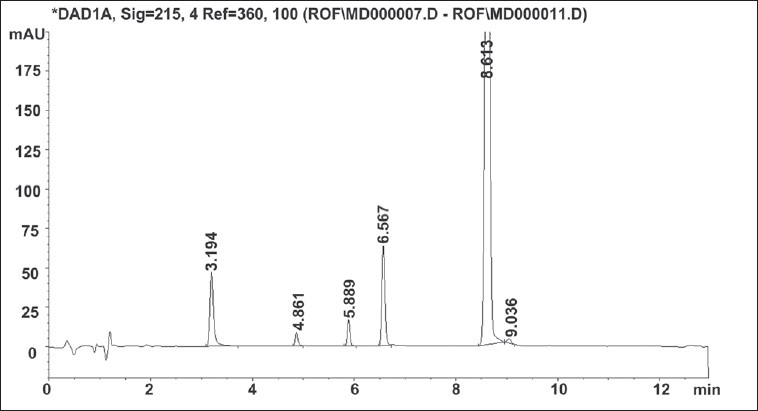
Chromatogram of alkaline degradation of roflumilast sample.

**Fig. 4:**
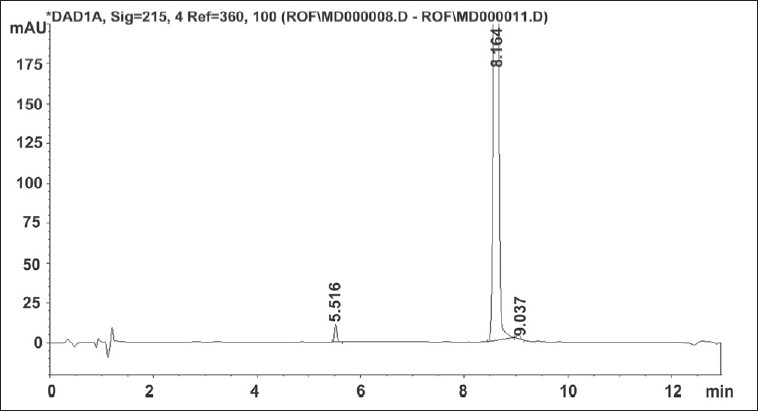
Chromatogram of acidic degradation of roflumilast sample.

**Fig. 5:**
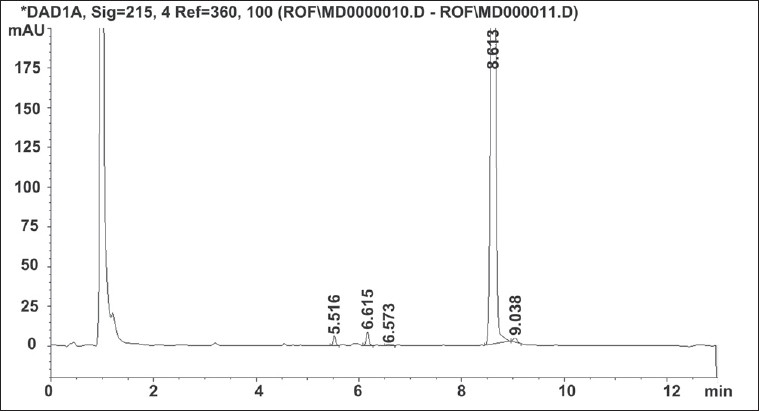
Chromatogram of oxidative degradation of roflumilast sample.

To study the effect of thermal stress, roflumilast was exposed to dry heat at 80° in a hot air oven for 24 h. Photostability of roflumilast was checked by exposing to UV light in the solid state[3]. Roflumilast was exposed to ≥200 W h m^-2^ UV irradiation at 320-400 nm, at 25° for 75 h. Each stressed sample was diluted with acetonitrile to achieve concentration of 150 µg/ml of roflumilast.

The maximum absorption wavelength of roflumilast solution and forcefully degraded drug solution was found to be 212 nm through their UV absorption spectra and hence, 215 nm was selected as detection wavelength for LC analysis.

Method development was done using conventional 250 mm column and a RRHT 50 mm, 1.8 µm column. A forced degraded solution of roflumilast was chromatographed on column packed with 5 µm reversed-phase C18 bonded stationary phase. Run time for gradient method was 38 min and roflumilast eluted at 18.6 min. HPLC run time was too long hence further method development was done using RRHT columns.

A mobile phase consisting of 0.1% formic acid and acetonitrile using gradient elution flowing at a rate of 0.5 ml/min was tried on 1.8 µm 50×4.6 mm RRHT columns[4]. Columns tried were, Zorbax Eclipse plus, Zorbax Extend C18, Zorbax XDB C18 and Zorbax SB C18. Better separation was achieved on Zorbax SB C18 50×4.6 mm, 1.8 µm as compared to other columns hence further experimentation was done using this column.

Replacement of acetonitrile by methanol resulted in merging of two degradation peaks observed in alkaline degradation of roflumilast. A gradient run using 0.1% formic acid and acetonitrile with organic concentration varying from 10 to 90% of aqueous phase over 20 min eluted the roflumilast at 11.1 min. The gradient run was modified to have short run time of 13 min. Other buffers of 0.005 M strength tried were ammonium formate pH 3.5, phosphate buffer pH 2.4[5], mixed phosphate buffer pH 6.6[5], ammonium acetate buffer pH 5 and ammonium bicarbonate buffer pH 7. Aqueous phase of 0.1% formic acid solution was too low in buffer capacity hence not considered for further method development process. Change in aqueous phase exerted imperceptible effect on retention time and peak shape of roflumilast. Comparison of various aqueous phases is shown in Table 2. Mobile phase pH from 2.4 to 7 did not have any influence on the retention time of roflumilast. Ammonium formate buffer pH 3.5 was preferred over other buffers due to less interference and its volatile nature as this can also be used for a LCMS method. The forced degradation behaviour of roflumilast under different forced degradation conditions is summarized in Table 3. Stress testing showed that all degradation products were well separated from roflumilast, confirming its stability-indicating capability.

**TABLE 2:** AQUEOUS PHASE COMPARISON

Aqueous phase	pH	Retention time of roflumilast (min)	Comments
Phosphate buffer	2.4	8.75	Interference in blank
Ammonium formate	3.5	8.6	Good separation between all degradation peaks and rofl umilast.
Ammonium acetate	5.0	8.33	Close elution of two degradation peaks in alkaline condition.
Mixed phosphate	6.6	8.27	Good separation between all degradation peaks and rofl umilast. But not suitable for LCMS.
Ammonium bicarbonate	7.0	8.31	Interference in blank

**TABLE 3:** FORCED DEGRADATION BEHAVIOR OF ROFLUMILAST

Condition	Time (h)	Temp (°)	% degradation	Area % of major degradation products						
			(RRT)→	0.37	0.56	0.64	0.68	0.72	0.76	1.05
Initial	0	0.62	-	-	-	-	-	-	-	0.62
0.1 N HCl	24	80	1.84	-	-	1.26	-	-	-	0.58
Water	24	80	0.63	-	-	-	-	-	-	0.63
0.1 N NaOH	24	80	18.4	7.02	0.91	-	1.71	-	8.21	0.55
30% H2O2	24	80	2.55	-	-	0.67	-	1.1	0.11	0.67
Thermal	24	80	0.62	-	-	-	-	-	-	0.62
UV light	75	25	0.61	-	-	-	-	-	-	0.61

All stressed samples, both solid and in solution, remained colourless. Forced degradation results prove that roflumilast is stable under neutral condition while in 0.1 N hydrochloric acid it shows low degradation. The rate of hydrolysis of roflumilast was found to accelerate in alkaline condition resulting in 18.4% degradation. Under oxidative condition, roflumilast shows three unknown impurities having major impurity of 1.1% at RRT 0.72. The drug was stable to thermal and photodegradation as indicated by no increase in the area of impurity peak.

A simple, economic and rapid HPLC analytical method with UV detection has been developed for the determination of roflumilast active pharmaceutical ingredient using a Zorbax SB C18 50 mm × 4.6 mm column with 1.8 µm particle size. The low run time of 13 min enables rapid determination of the drug, which is important for the routine analysis. A method was also developed on a conventional C18 250×4.6 mm column wherein run time was 38 min.

The method seems to be suitable for the quality control in the pharmaceutical industry because of its sensitivity, simplicity, selectivity and short run time. Time required for the analysis of 50 injections using 38 min run time method on 250×4.6 mm column will be approximately 31.6 h. The same analysis can be completed in 11 h using 13 min run time method on 50×4.6 mm column packed with 1.8 µm particles excluding column equilibration time for both the columns. This indicates use of 1.8 µm short column offers several advantages such as low run time, higher productivity and low solvent consumption resulting in cost reduction and being environment friendly.
